# Beyond the Backbone: The Next Generation of Pathwalking Utilities for Model Building in CryoEM Density Maps

**DOI:** 10.3390/biom12060773

**Published:** 2022-06-02

**Authors:** Corey F. Hryc, Matthew L. Baker

**Affiliations:** Department of Biochemistry and Molecular Biology, Structural Biology Imaging Center, McGovern Medical School, The University of Texas Health Science Center, 6431 Fannin Street, Houston, TX 77030, USA; corey.f.hryc@uth.tmc.edu

**Keywords:** cryoEM, Pathwalking, de novo modeling, near-atomic resolution, density map, probabilistic models, ligand modeling

## Abstract

Single-particle electron cryomicroscopy (cryoEM) has become an indispensable tool for studying structure and function in macromolecular assemblies. As an integral part of the cryoEM structure determination process, computational tools have been developed to build atomic models directly from a density map without structural templates. Nearly a decade ago, we created Pathwalking, a tool for de novo modeling of protein structure in near-atomic resolution cryoEM density maps. Here, we present the latest developments in Pathwalking, including the addition of probabilistic models, as well as a companion tool for modeling waters and ligands. This software was evaluated on the 2021 CryoEM Ligand Challenge density maps, in addition to identifying ligands in three IP_3_R1 density maps at ~3 Å to 4.1 Å resolution. The results clearly demonstrate that the Pathwalking de novo modeling pipeline can construct accurate protein structures and reliably localize and identify ligand density directly from a near-atomic resolution map.

## 1. Introduction

Once restricted to relatively low resolutions, single-particle electron cryomicroscopy (cryoEM) has emerged as a powerful structural biology tool, capable of deciphering complex functional mechanisms in large, complex macromolecular assemblies [1,2,3]. The rapid adoption of cryoEM has been primarily due to technological advancements in electron microscopes, imaging hardware, and tools for data processing that have made solving structures at near-atomic resolution routine [4]. Furthermore, new computational tools have been developed to enable reliable atomic model generation, even in complex density maps at varying resolutions [5,6]. Today, cryoEM is at the forefront of biomedical science and human health, providing atomic detail for the structure and function of ribosomes [7], ion channels [8], G-protein coupled receptors [9], CRISPR [10], and SARS-CoV-2 [11].

While density maps at near-atomic resolutions were relatively uncommon a decade ago, it is now common to achieve better than 4 Å resolution with single-particle cryoEM [12]. State-of-the-art structures are even pushing toward 1 Å resolution and can resolve densities for individual atoms [13,14,15,16]. As such, computational modeling tools for analyzing and generating models from cryoEM density maps have also become standard fare [17].

Originally, model building in cryoEM was restricted to feature-recognition methods such as secondary-structure element identification [18], template-based rigid body fitting [19], flexible fitting [20] or density-constrained modeling [21]. In recent research, new fitting tools leverage predictive modeling via AlphaFold2 and RosettaFold for generating models from cryoEM density maps (https://doi.org/10.1101/2022.01.07.475350). While these methods play critical roles in our understanding of macromolecular structure and function, today’s most common methods for analyzing near-atomic resolution density maps focus on building models directly from the map itself. The first “de novo” modeling approaches adapted feature recognition tools, where secondary structures in the map and those predicted from the sequence were used to derive a protein fold [22], though with improving resolutions, new computational tools have looked to exploit “high-resolution” features in maps to build all-atom models [6]. These methods, coupled with manual and automated refinement tools to optimize chemistry and density fitting [23,24,25], have resulted in cryoEM models with similar quality to those from X-ray crystallography and NMR.

Most of the current modeling tools are based on a similar approach; visible features in the density map are used to establish a basic fold, from which a variety of force fields and chemical restraints help place and refine atom positions [26]. In Phenix, automated modeling building is accomplished by generating a range of atomic models using several independent methods followed by real-space refinement with restraints [27]. MAINMAST generates a set of points, determines and refines connections among these points, and then threads a sequence onto the trace for subsequent refinement [28]. With DeepTracer, 3D deep learning methods are used to rapidly construct atomic models directly from cryoEM density maps [29]. Regardless of the approach, these procedures are relatively robust and can build reliable models at resolutions better than 2.5 Å, though some require large computational resources [6]. At lower resolutions, or in density maps with varied resolvability, “standard” approaches tend to fall short.

With Pathwalking, we reimagined model building as a geometrical optimization problem bound by chemical constraints [30,31,32]. Pathwalking is based on the traveling salesperson problem (TSP); TSP solvers calculate possible cyclical paths between a set of nodes, minimizing the distance traveled [33]. Here, the TSP solver is used to calculate a path, i.e., the protein fold, using a set of derived nodes (Cα atoms) from a target cryoEM density map without explicitly using sequence or structural constraints. The only required input for Pathwalking is a density map (<5 Å resolution) and the number of amino acids in the protein. Iterative path optimization with increasing physical constraints is used to find an optimal path through the density. Once an optimal path is found, the sequence is threaded onto the path, optimized to fit density, and refined against chemical constraints.

As with other cryoEM modeling tools, there are a few shortcomings that can limit the ability to build reliable, robust models in cryoEM density maps [5,6]. First, modeling non-protein density, including nucleic acids, waters, ions, small molecules, and other ligands is generally carried out by hand and only included during the final model refinements. Second, differences in resolution and resolvability, common in cryoEM density maps, may result in varying model quality between, or even within, individual subunits. While there are a number of methods to assess model quality [34,35], there are almost no mechanisms that simultaneously assess model degeneracy in terms of both density fitting and chemistry.

In this study, we addressed two major bottlenecks in de novo model building with Pathwalking. In addition to several algorithmic and automation improvements, we implemented a mechanism to rapidly generate alternate paths from the density map and compare these paths in an effort to determine an accurate probability score for model paths. Furthermore, we designed a new companion tool, pw_ligands.py, that builds on a number of the core Pathwalking concepts, to localize and identify waters, ions, and other ligands. Together, these tools offer a more robust and comprehensive de novo modeling platform for near-atomic resolution density maps.

## 2. Materials and Methods

### 2.1. Workflow Overview

**Figure 1:**
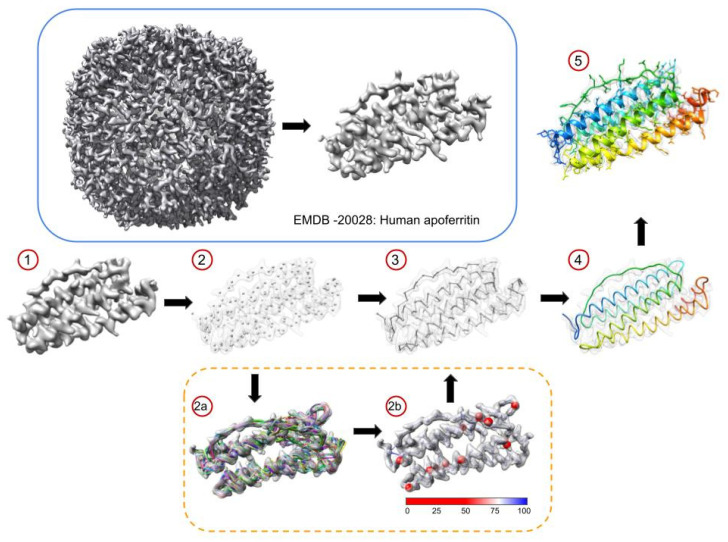
Pathwalking. The basic steps of Pathwalking using EMD-20028, human apoferritin at 3.1 Å resolution, are shown. In the blue box, the reconstruction of apoferritin is shown with a single monomer segmented from the density map. In (**1**), the map is low-pass-filtered to ~4.5 Å resolution, which is then followed by pseudoatom generation (**2**). An initial trace is computed using a TSP solver in (**3**) and then refined to optimally fit the density and remove any non-protein-like features (**4**). Finally, the sequence is threaded onto the model, from which a full atom model is generated and real-space-refined using the original segmented density map (**5**). The new version of Pathwalking has the option to generate a set of decoy models from which a probabilistic model can be calculated (dashed orange box). In (**2a**) 100 separate paths over 5 thresholds and 20 noise levels were calculated using Pathwalking. In (**2b**), an average model from the 100 paths is shown. Connections are colored based on probability that pseudoatom N is connected to N + 1 and N − 1 in all 100 models. Red spheres indicate instances where the connection probability was below 50%.

The set of Pathwalking tools for de novo model building in cryoEM density maps can be summarized in five basic steps: (1) map filtration, (2) pseudoatom generation, (3) path identification, (4) path optimization, and (5) sidechain assignment and refinement (Figure 1). Each of these steps is critical in the generation of the final model, though the pseudoatom generation, path identification, and path optimization steps, unique to Pathwalking, are the key determinants of successful de novo modeling.

In an effort to unify the Pathwalking utilities and provide for improved developer access, we completely rewrote the original software in Python3 and incorporated it into Phenix, a comprehensive software package for macromolecular structure determination (for more details, see Supplementary Methods) [36]. An updated list of all Pathwalking options can be found in Table 1. Pathwalking and the new companion tool, pw_ligands.py, are now available via the command line in Phenix. This step was critical in providing the framework for the new features discussed below.

### 2.2. Probabilistic Models

On a modern laptop or desktop computer, Pathwalking takes a few seconds to generate a potential backbone trace for typically sized proteins. With this in mind, it is possible to generate a large set of potential paths and compare these paths in a fraction of the time that it takes other de novo modeling methods to generate a single model. Once a set of potential paths are generated, it becomes feasible to statistically analyze the collection of paths and determine probabilities for connectivity at each point along the path. Collectively, these paths (decoys) and the connection probabilities between each point along a path can be used to generate a probabilistic model and quantitatively assess the reliability of the model at each pseudoatom position.

In the current version of Pathwalking, we have implemented an option to allow users to generate probabilistic models directly from the density map (dashed orange box in Figure 1). As described, Pathwalking begins with the population of pseudoatoms in the density; the pseudoatoms offer a reduced representation of the density map and serve as the nodes used in the TSP solver. Here, the user provides a set number of pseudoatoms (corresponding to the number of Cα atoms in the map) and a single map threshold, where the overall connectivity of the density map can be observed. In generating a probabilistic model, several sets of pseudoatoms are computed across a range of thresholds rather than a single density threshold, though each set contains the same number of pseudoatoms. Additionally, for each set of pseudoatoms, small positional perturbations are introduced, creating a “library” of pseudoatom positions based on different density thresholds with various amounts of positional noise. The number and range of thresholds, as well as the positional noise and number of alternate positions, can be defined by the user, making it possible to generate tens to thousands of sets of pseudoatoms.

Each set of pseudoatoms is used as an input to the TSP solver, whereby the optimal backbone path can then be constructed. The set of all paths, or “decoys”, are then aligned, renumbered, and compared. While the exact path of each of these decoys will vary, the total number of pseudoatoms generated remains constant and in the same coordinate system as the map of interest. From the set of decoys, an average position for each point is calculated, creating an “average” set of pseudoatoms; the path for this set of pseudoatoms is then calculated using the Pathwalking TSP solver. All decoys are then compared with the average model path. A simple percentage is calculated comparing the connectivity of each pseudoatom, N, in the average path to the connectivity of the corresponding pseudoatom in each of the decoy paths. As the paths from Pathwalking are agnostic to direction, both the N + 1 and N − 1 positions are examined. This probability value is then mapped to the B-factor column in the PDB file, whereby it can be displayed graphically in molecular visualization software such as UCSF’s Chimera and ChimeraX [37,38].

### 2.3. Modeling Waters and Other Ligands

**Figure 2:**
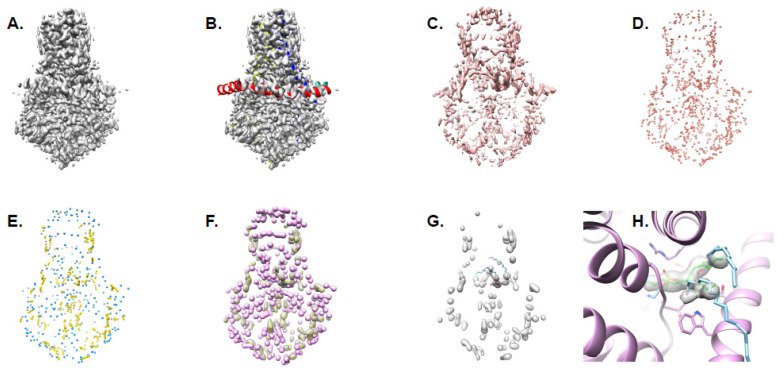
Ligand identification. The steps for generating a full-atom model complete with ligands using Pathwalking and pw_ligands.py is shown in order: (**A**) map filtration, (**B**) Pathwalking, (**C**) masking out of protein density, (**D**) generation of pseudoatoms (salmon) in non-protein density, (**E**) filtering pseudoatoms into putative ligands (yellow) and waters/ions (blue), (**F**) creating density masks for putative waters (purple) and ligands (yellow), (**G**) fitting ligand to density, and (**H**) refinement of ligand into density with protein components. The steps in panels (**C**–**F**) are automated with pw_ligands.py, while steps in panels (**A**,**B**) are part of Pathwalking, ligand docking (**G**) is performed in Chimera or Coot and ligand refinement (**H**) is accomplished with Phenix.

Typically, only proteins are considered during de novo modeling in near-atomic resolution density maps, though waters and other ligands are often found in the maps. To incorporate ligand and water modeling into the Pathwalking pipeline, we utilized our pseudoatom representation of the density map to effectively locate and identify these components (Figure 2). At the outset of model building, the population of pseudoatoms occurs primarily in regions of the protein backbone. Once a preliminary protein model is constructed using Pathwalking, the protein-associated density can then be masked and a new set of pseudoatoms populated in the remaining map. These pseudoatoms correspond to the non-protein components, though they must be filtered, as the density map may contain “noise” from image processing and reconstruction. To this end, distance from the model (user definable, default <5 Å) and map resolvability, based on the consistency of voxel values in half-maps (user definable, <50% voxel value difference), are used to eliminate pseudoatoms from “noisy” density. Once filtered, size and shape analysis is performed to characterize the regions of interest. The location and shape of a pseudoatom distribution are then used to interpret regions of interest and assign them as either an ion/water or ligand. Once regions are classified as either waters/ions or ligands, structural templates for the corresponding features can be fit to the pseudoatoms distributions, and their fit to the density measured using standard correlation-based techniques. A complete list of the pw_ligands.py arguments and options are listed in Table 2.

### 2.4. Evaluation and Assessment of Pathwalking

The latest version of Pathwalking, complete with ligand identification and probabilistic models, was used to process the 2021 CryoEM ligand Challenge dataset (https://challenges.emdataresource.org/?q=2021-model-challenge, accessed on 1 January 2022). Each of the three datasets contained multiple protein chains and various ligands, all of which were visible in the 1.9–2.5 Å resolution maps. Individual protein subunits were first segmented using Segger in UCSF’s Chimera.

Models for each protein were constructed using mostly default Pathwalking options: a segmented density map, map threshold, the number of expected amino acids, refinement resolution, a sequence file, and the all-atom flag were specified for each protein subunit modeled. Additionally, the flags for map filtering and probabilistic models, including threshold bracketing (threshold, +/− 1 sigma) and positional noise (20 values from 0 to 5 sigma), were specified. The final Pathwalking models for the individual subunits were then assembled into individual complexes and refined using the default options in phenix.real_space_refine; non-crystallographic symmetry (NCS) constraints were applied if the maps contained symmetry.

For ligand identification, the original density map, the protein-only model, and a density threshold at which ligands can be seen were provided as input. Unlike Pathwalking, the number of pseudoatoms is not explicitly specified and is computed automatically. Additionally, half-maps, which are used in pseudoatom filtering, were provided as inputs when available. The results from the pw_ligands.py program were two coordinate files and two density maps, one each representing potential ligands and the other set representing waters and ions. The ligand density map was loaded into UCSF Chimera, along with a coordinate file for the appropriate ligands. The ligands were then fit to the ligand map with the Fit to Density function in Chimera. The coordinates for the ions and waters were then loaded into Chimera. A final model containing the protein, ligands, and water components was then saved and refined once with phenix.real_space_refine.

For the 2021 CryoEM Ligand Challenge datasets, evaluation of model quality, assessment of fit to density, and comparison to the reference structure were assessed independently using the model comparison pipeline (https://model-compare.emdataresource.org/2021/cgi-bin/index.cgi, accessed on 1 January 2022). Additionally, Phenix was used to generate relevant statistics.

While not included in the probabilistic model generation evaluation, ligand identification was run on three slightly lower resolution datasets from the inositol-1,4,5-trisphosphate receptor (IP_3_R1) reconstructions: a 2.96 Å resolution structure of apo-IP_3_R1 reconstituted with LMNG (EMD-23337, PDB ID: 7LHF), a 3.3 Å resolution structure of apo-IP_3_R1 in nanodisc (EMD-2333, PDB ID: 7LHE) [39], and a 4.1 Å resolution reconstruction of IP_3_R1 bound to adenophostin A (EMD-7770, PDB ID: 6MU1) [40]. Evaluation of ligand identification in the IP_3_R1 models was carried out by comparing the results to the published models in Chimera.

## 3. Results

### 3.1. Overall Results

As seen in Table 3, automated Pathwalking on the three CryoEM Ligand Challenge datasets produced accurate and robust structures for the individual protein complexes. All three Pathwalking models had minimal clashes, Ramachandran outliers, and rotamer outliers, as well as Molprobity scores below 2.0, suggesting that model quality for these models was at least on par or better than the reference model. In addition to model quality, density fitting for each of the models was assessed. As with model quality, all density-fitting metrics, including cross-correlation, Fourier shell correlation (FSC), atom inclusion, Q-scores [41], and EMRinger scores [34], indicated that both the mainchain and sidechain atoms fit the density well. Moreover, each of the three models agreed well with the reference structure, having less than 1 Å RMSD and near-ideal LDDT and GDT_TS scores. Together, these results indicate that Pathwalking can indeed produce highly accurate models that can capture all of the protein density features in this resolution range.

### 3.2. Probabilistic Model Results

**Figure 3:**
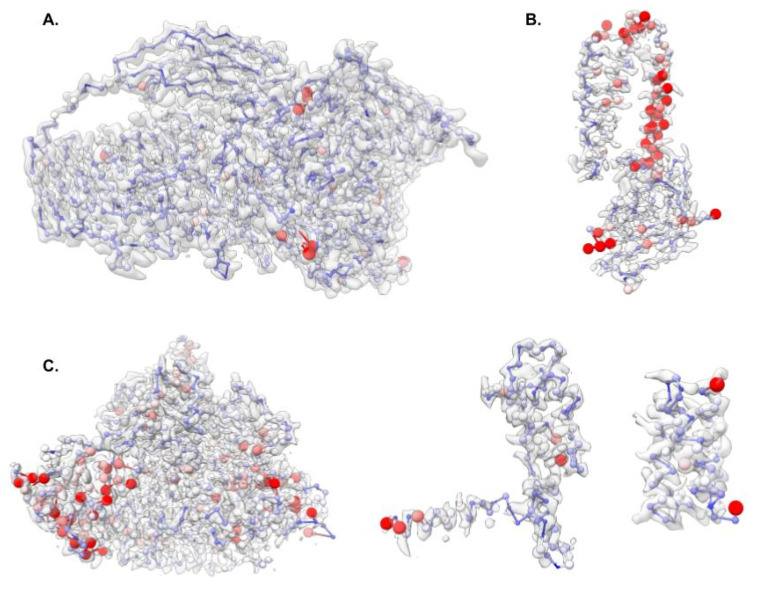
Probabilistic models for the 2021 CryoEM Ligand Challenge datasets. Probabilistic models for unique individual protein subunit are shown for EMD-7770 (**A**), EMD-22898 (**B**) and EMD-30210 (**C**). In (**A**), only one of the four β-galactosidase subunits is shown. In (**C**), each panel represents one of the three protein chains (A, B and C) in the map. All models are colored based on connection probability from 100% (blue) to 75% (white) and 50% or below (red). Additionally, atom diameter reflects the reliability of connectivity with larger atom diameters having less confidence.

For each of the protein components in the three CryoEM Ligand Challenge datasets, probabilistic models were also generated across three thresholds (visually identified value and +/− 1 sigma) and 20 noise levels ranging from 0 to 5 sigma for a total of 60 independent paths. On an Intel Apple Mac Mini, the generation of each set of models ranged from ~5 min (EMD-30210, chain C) to ~30 min (EMD-7770, chain A). The probabilistic model (i.e., the average path with connectivity probabilities) for each subunit was visualized in UCSF’s Chimera (version 1.16) and colored based on connection probability to identify regions of potential model variability (Figure 3).

The overall fold of the probabilistic model for each of the protein subunits in the CryoEM Ligand Challenge datasets revealed nearly identical folds to the published models; each model had a TM-score [42] of at least 0.9 when compared with the published model. Further examination of the decoy models used in the generation of the probabilistic models revealed some common themes in potential modeling errors. The most common connectivity issue seen was related to the termini, where noise and threshold perturbations resulted in connectivity differences among the first two and last two pseudoatoms, resulting in lower connectivity probabilities at the termini. Similarly, swapping the order of connectivity among two or three amino acids was seen among the decoy models. This is due to the noise and threshold perturbations altering the optimal order of local connectivity among a small group of points. Interestingly, these local variations were not restricted to secondary structure type, as this was seen throughout the models, including β-strands in EMD-7770 [43], an α helix of EMD-22898 [44], and loops in EMD-30210 [45]. In all cases, some of the models with these errors had a less “protein-like” path and a higher potential for alternate connectivity between small groups of atoms, though the overall fold of the protein was not changed. Regardless, the probabilistic models for each of the datasets were accurate and provided a new measure of reliability for Pathwalking and model connectivity.

### 3.3. CryoEM Ligand Challenge Results

**Figure 4:**
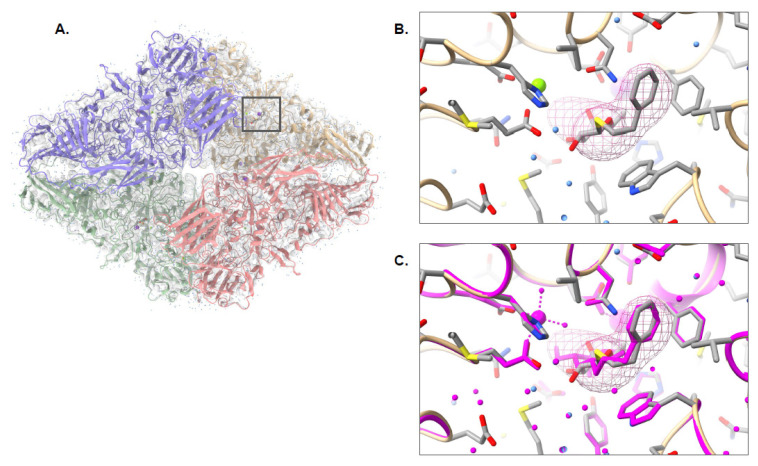
Pathwalking and ligand identification: EMD-7770. The complete Pathwalking model for β-galactosidase, including water (blue) and ligands (gray) is shown in (**A**). A zoomed-in view of the identified ligand location, marked by a square in (**A**), by pw_ligands.py is shown as a cage in (**B**). The fitted and refined ligand, along with the water (blue) and protein models (colored by chain) are shown as well. In (**C**), the published model (magenta) is shown superimposed on the Pathwalking model.

Once the models for the three 2021 CryoEM Ligand Challenge datasets were constructed, pw_ligands.py was used to locate potential ligands and waters in the corresponding density maps. In EMD-7770, the density corresponding to the 2-phenylethyl 1-thio-beta-D-galactopyranoside (PTQ) molecule in the published structure (PDB ID: 6CVM) was localized in each of the four subunits of the β-galactosidase density map. A single PTQ model was then fit into the density and refined along with the Pathwalking model into the density map. In Figure 4, the fit PTQ molecule can clearly be seen occupying the same position as the PTQ molecule in the published structure. The resulting 0.2 Å RMSD between the model and the known structure, as well as a Q-score of 0.85, further reinforces the accuracy to which the ligand density was identified and modeled. Likewise, pw_ligands.py identified 2608 potential water positions; the published structure contained 4194 potential water positions. Of the 2608 water positions, 1814 identified water positions were within 1 Å of waters in the published structure and only 356 of the waters were further than 5 Å away from a corresponding water position in the published structure.

**Figure 5:**
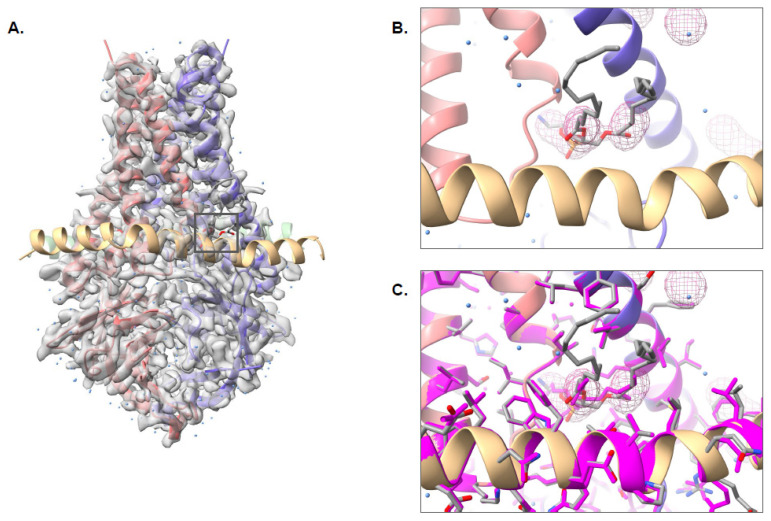
Pathwalking and ligand identification: EMD-22898. The complete Pathwalking model for SARS-CoV-2 ORF3a putative ion channel in nanodisc, including waters (blue) and ligands (gray) is shown in (**A**). A zoomed-in view of the identified ligand location, marked by a square in (**A**), by pw_ligands.py is shown as a cage in (**B**). The fitted and refined ligand, along with the water (blue) and protein models (colored by chain) are shown as well. In (**C**), the published model (magenta) is shown superimposed on the Pathwalking model.

As with EMD-7770, the structure of SARS-CoV-2 ORF3, a putative ion channel in nanodisc (EMD-22898), was accurately modeled with Pathwalking; The RSMD between the model and the known structure (PDB ID: 7KJR) was less than 1Å, and the LDDT score was 1.00 (Figure 5A). pw_ligands.py also correctly identified the 1,2-dioleoyl-sn-glycero-3-phosphoethanolamine (PEE) site in the density map. Interestingly, additional density for the tails of PEE was also identified with pw_ligands.py, and as a result, more of the PEE tails could be modeled. Automated fitting and refinement of the ligand resulted in a Q-score of 0.71 for the ligand; the fit of PEE is nearly identical between the published structure and pw_ligands.py (Figure 5B,C). Additionally, 178 putative waters were localized in the density, though only 122 were present in the published structure. Of the 178 waters, 71 were within 1 Å and 21 were greater than 5 Å away from a corresponding water position when comparing waters identified by find_ligand.py and the published structure.

**Figure 6:**
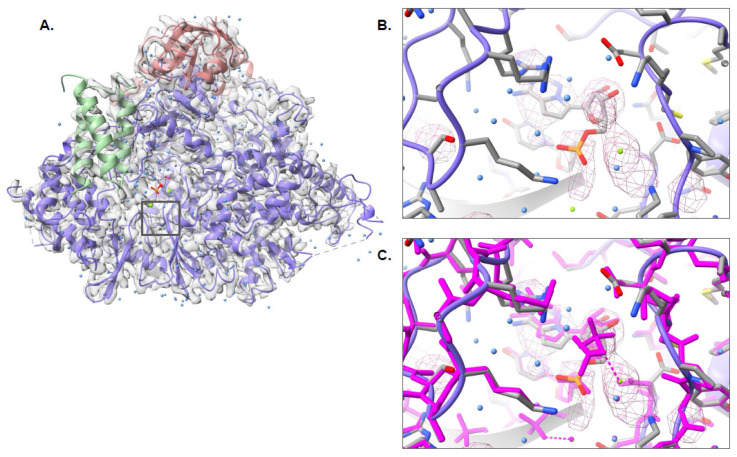
Pathwalking and ligand identification: EMD-30210. The complete Pathwalking model for SARS-CoV-2 RNA-dependent RNA polymerase, including waters (blue) and ligands (gray) is shown in (**A**). A zoomed-in view of the identified ligand location, marked by a square in (**A**), by pw_ligands.py is shown as a cage in (**B**). The fitted and refined ligand, along with the water (blue) and protein models (colored by chain) are shown as well. In (**C**), the published model (magenta) is shown superimposed on the Pathwalking model.

The third dataset from the 2021 CryoEM Ligand Challenge was the SARS-CoV-2 RNA-dependent RNA polymerase, EMD-30210, which contained F86 (remdesivir) and RNA. At this time, Pathwalking does not model nucleic acids and, as such, construction of the RNA was carried out manually using Coot. As with the other two challenge datasets, Pathwalking was used to build a model of the protein components, followed by ligand identification with pw_ligands.py (Figure 6A). The overall model had an RMSD of 0.37 Å and an LDDT score of 0.96 when compared with the published model (PDB ID: 7BV2), indicating the model was constructed with high fidelity. pw_ligands.py identified the F86 site in the density map correctly and the ligand was automatically fit to the ligand density and refined with the full model (Figure 6B,C). The resulting fit of the ligand had a Q-score of 0.79 and almost perfectly replicated the reference fit of F86 into the density map. A total of 266 putative waters were identified on the map, though only 5 were reported in the published structure. All five of the published water locations were within 1 Å of a water location identified using pw_ligands.py.

### 3.4. Ligand Identification at Lower Resolutions

**Figure 7:**
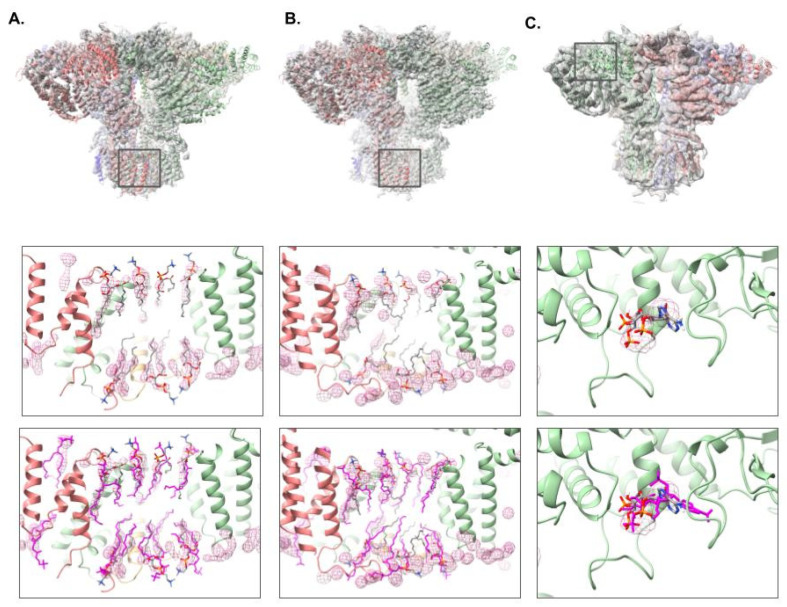
Ligand Identification in IP_3_R1. The Pathwalking models with ligands identified using pw_ligands.py from three different IP_3_R1 reconstructions are shown, Results from the 3.3 Å and 2.96 Å IP_3_R1 reconstructions are shown in (**A**) and (**B**), respectively. In (**C**), the 4.1 Å reconstruction of IP_3_R1 contains the ligand, adenophostin A (ADA). The identified ligand density and lipids are shown in the middle panel, while the model for the published structure (magenta) is shown in the lower panel. A zoomed-in view of the identified ligand region, marked with a square in the top row, can be seen in the middle panel. In all three of the models, only a single set of lipids are shown per tetramer.

Beyond the 2021 CryoEM Ligand Challenge datasets, three ligand-bound datasets of IP_3_R1 at various resolutions were also used to assess the effectiveness of the ligand identification. In the 3.3 Å and 2.96 Å resolution IP_3_R1 density maps, pw_ligands.py was able to clearly identify seven unique regions per subunit that were not modeled as part of the protein components (Figure 7A,B). Each of the seven regions contained a globular region accounting for the head group of a lipid and at least one protruding tail-like density. Seven phosphatidylcholine molecules were docked into the identified ligand densities and refined with phenix.real_space_refine. All seven of the automatically identified ligands in these two density maps matched up well with the ligands found in the corresponding published structures, suggesting that our automated ligand identification tool works well in ~3 Å resolution maps.

While phosphatidylcholine molecules were not previously detected in the 4.1 Å resolution density map of IP_3_R1, the reconstruction did contain adenophostin A (ADA) in the IP_3_ binding pocket between the ARM1 and β-TF1/β-TF2 domains. As with the previous examples, pw_ligands.py was used to localize the ligands in this density map. The ADA density was clearly identified in the IP_3_ binding pocket and docking of the ADA molecule produced a nearly identical fit of ADA when compared with the published structure (Figure 7C). Additionally, some potential ligand density was observed in the region corresponding to the phosphatidylcholine in the other maps. However, no clear tail and only partial density for the head groups were observed, and as such, these densities were not modeled.

### 3.5. Computing Times and Environment

As described, one of the major advantages of Pathwalking is its ability to quickly obtain high-quality models. For all aforementioned maps, Pathwalking and ligand identification were carried out on a 6-core 3.2GHz Intel Core i7 Apple Mac Mini with 32GB of DDR4 RAM (2018), though any relatively modern desktop or laptop computer running Windows, Linux, or macOS is capable of running the software. Automated model generation using default Pathwalking options for the three CryoEM Ligand Challenge datasets took ~0.5–1 h per subunit. The addition of probabilistic models added between 5 and 30 min to the computing of the final, refined Pathwalking model. Ligand identification, docking, and refinement required an additional ~0.5–1 h of computing time. These times are fairly typical in our experience on these and a variety of other maps.

Diving deeper into the compute times, the major bottlenecks are actually in setting up the TSP search and final model refinement. With Google OR tools, the default TSP solver in the latest version of Pathwalking, the maximum path search time is capped at 30 s though optimal solutions are usually found much quicker. Depending on the number and which pseudoatom method is specified, seeding pseudoatoms in the map takes between 30 s and 5 min for most proteins. Real-space refinement of the entire model is the most time-consuming step and is largely dependent on map size but typically averages 10–15 min when utilizing the default options.

## 4. Discussion

In an effort to provide the most robust set of modeling tools for all near-atomic resolution density maps, we have continued to innovate and improve our Pathwalking utilities. These improvements, which include a number of algorithmic updates, as well as options for generating a more statistical approach for assessing model reliability, have allowed for an almost completely automated approach to modeling protein structures. Moreover, these new improvements, while adding a small amount of computational time, also allowed us to incorporate water and ligand identification into the Pathwalking model building pipeline. As seen in the evaluation of our tools with the 2021 Ligand Modeling Challenge datasets, model building, assessment, and ligand identification required only limited computational resources to generate highly accurate models. A summary of the results in Table 3 indicated that all models had the same fold and low RMSDs (<1 Å) when compared with their corresponding published structures.

### 4.1. Probabilistic Models

The new probabilistic models use a simple mechanism to assess the connectivity of neighboring pseudoatoms across multiple models. The simple and straightforward approach of generating multiple models across a series of thresholds and noise levels leverages the speed of the TSP solver, which can effectively compute an optimal path through a large set of points in a few seconds. As such, generating a large gallery of potential models and assessing their agreement for constructing a probabilistic model is easy and requires only several minutes to an hour to generate hundreds of possible models. While the generation of a large number of decoy models would likely improve statistical sampling, our empirical results suggest that only 50–100 models are required to gain a meaningful estimation of connectivity probability for a typically sized protein.

It should be noted that the models generated from the three CryoEM Ligand Challenge datasets using Pathwalking with and without probabilistic models were nearly identical after real-space refinement. While the probabilistic models do not necessarily guarantee an improvement in final model accuracy, they provide a simple mechanism for identifying and tracking potential modeling issues, from the initial path tracing through final model refinement. As shown in Figure 3B (EMD-22898), several of the decoy models had pseudoatoms placed in bulky sidechain density along one of the major helices, resulting in poor geometry and degenerate models along the helix. While easily fixed during subsequent all-atom refinements, the probabilistic model for EMD-22898 provided a visual means to assess path quality, spot the potential issue, and monitor refinement results.

In future versions of Pathwalking, the identification of alternate paths with probabilistic models could play a more direct role in pseudoatom reseeding and model generation, as well as a potential mechanism for local rebuilding in predictive modeling with cryoEM density maps. As there are new options in Pathwalking to load a precomputed set of pseudoatoms, our probabilistic modeling could easily be applied to models generated using other cryoEM modeling tools. The only caveats to this would be that the input model would need to be a Cα-only model and be in the same coordinate system as the corresponding map. As such, the probabilistic models could be used as a common metric for assessing the reliability of any cryoEM model, regardless of the program used to generate the model.

### 4.2. Ligand Identification

While not as automated as Pathwalking, the ligand identification tool, pw_ligands.py, provides a semi-automated approach for accurately detecting ligand density in near-atomic resolution density maps. Based on a comparison with the published structures, pw_ligands.py was able to correctly detect the location of various ligands in the aforementioned density maps. For the 2021 Ligand Modeling Challenge datasets, pw_ligands.py performed similarly, and in some cases better than other more computationally intensive ligand identification and fitting tools. Beyond the ~2 Å resolution Ligand Modeling Challenge datasets, ligand identification was successful in the 3–4 Å resolution maps of IP_3_R1. We believe the maps presented here are typical examples of their reported resolutions and, as such, pw_ligands.py should perform equally well on similar resolution maps. As part of ongoing work, we are further automating the fitting and refinement of the ligand into the density map; future updates of the software with this feature will appear in Phenix.

An important point to consider in the identification of waters and ligands with pw_ligands.py is the relationship between resolution and resolvability. Our tool for finding ligand density is limited by the ability to distinguish ligands from protein density. As such, ligand identification is an issue of resolvability and not necessarily resolution. While we tested various datasets from 1.9 to 4.1 Å resolution, the success of pw_ligands.py was based on the fact that density associated with putative ligands could be reliably observed and quantified. Poorly resolved maps, anisotropic resolution, noisy density maps, and partial ligand occupancy could all lead to inaccurate ligand assignments. As such, it is difficult to assign an exact resolution range for the pw_ligand.py tool without considering all factors that go into the reconstruction.

Having said that, due to the expected size of the corresponding density, the identification of waters based on size and shape may not be reliable at resolutions worse than 3 Å. In maps better than 3 Å resolution, it is sometimes possible to see coordinated or bulk waters in the reconstructions, at which point our pw_ligands.py tool can localize the putative waters. It should be noted that pw_ligands.py did locate several potential water sites in the IP_3_R1 reconstructions, though at the reported resolutions, these sites were not distinguishable from noise in the reconstruction and thus, not reported in the final models.

In the 2021 Ligand Modeling Challenge datasets, all of which had resolutions better than 3 Å, our pw_ligands.py tool identified more waters than were present in two of the three published structures. In the third dataset, pw_ligands.py identified nearly two-thirds of the waters present in the published structure. What accounts for the discrepancies in the number of waters between the published models and those found by pw_ligands.py? Our ligand identification tool attempts to find un-modeled, non-protein density in the map, and part of this is achieved by examining the consistency of the voxel values between density half-maps computed during the reconstruction process. We surmise that this step is responsible for eliminating the bulk of potential waters. As an example, pw_ligands.py seeds over 8000 initial sites in EMD-7770, which were pruned to ~3000 putative ligand sites after filtering against the half-maps and proximity to the model. It is also interesting to note that pw_ligands.py does not explicitly use any chemical constraints to identify waters, ions, or ligands. Rather, identification is accomplished using statistical, geometrical, and shape constraints. Only at the time of refinement are modeled ligands subjected to chemical constraints. As such, regions with favorable ligand binding chemistry may be missed if the ligand density is poorly resolved. Given these constraints, the number of waters/ions may vary between our ligand identification tool and others. Interestingly, none of the submitted models for the 2021 Ligand Modeling Challenge reproduced the exact number of waters in the published structure. However, the vast majority of waters in the published structure agreed with those identified by pw_ligands.py. With the incorporation of Pathwalking and pw_ligands.py into Phenix, the addition of chemical constraints is the subject of ongoing work and will hopefully result in improved water identification and localization in cryoEM models.

## 5. Conclusions

The latest version of Pathwalking and its companion ligand identification tool, pw_ligands.py, have provided an improved approach to modeling large macromolecular complexes from near-atomic resolution density maps. Like its predecessors, these tools require minimal computational hardware while providing best-in-class performance. The inclusion of additional statistical measures of model accuracy and ligand identification fills a number of gaps in the previous version of the software and now provides a nearly completely automated system for cryoEM modeling.

## Figures and Tables

**Table 1:** Table of Pathwalking options. A list of all command-line inputs, along with a description, value type, and default setting is provided. Pathwalking is invoked using phenix.pathwalker <map file> <threshold> <nres => <options>.

Inputs	Required?	Default Value	Description
map_file	required	na	Density map of interest in CCP4 or MRC format.
Threshold = <float>	required	na	Threshold value at which resolution appropriate features can be seen.
nres = <int>	required	na	Number of pseudoatoms to generate in the density map—usually corresponds to the number of expected amino acids.
seq_file = <file>	optional	na	Text file containing the 1-letter amino acid sequence of the protein.
pa_file = <file>	optional	na	Loads previously generated pseudoatoms in PDB format.
verbose = <bool>	optional	False	Verbose output.
tsp = <>	optional	ortools	Select TSP solver: ortools or LKH.
pa_type = <>	optional	kmeans	Select pseudoatom generation method: kmeans, sc, ac, ms, gmm.
noise = <float>	optional	0	Adds stochastic noise to pseudoatom positions.
map_weight = <bool>	optional	False	Weight pseudoatom distances based on density map values.
filt = <bool>	optional	False	Apply a 4.5 Å low pass filter to the input map.
or_time = <int>	optional	30	Maximum time (seconds) for TSP solution calculation in OR tools.
prob_model = <bool>	optional	False	Calculates a probabilistic model when more than 1 path is computed.
all_atom = <bool>	optional	False	Converts the final model into an all-atom model; requires seq_file.
reverse = <bool>	optional	False	Threads the sequence file both forwards and backward on the path; requires seq_file.
refine_resolution = <float>	optional	0	Resolution used for real-space refinement.
bracket = [float,float,float]	optional	na	The minimum, maximum, and interval for specifying multiple thresholds.
tsp_runs = [float]	optional	0	A list of noise levels to apply to pseudoatom positions. A unique path is calculated for each TSP run. Can be combined with brack.

**Table 2:** Table of pw_ligands.py option. A list of all command-line inputs, along with a description, value type and default setting is provided. Pw_ligands.py is invoked using python pw_ligands.py <map file> <threshold> <nres => <options>.

Inputs	Required?	Default Value	Description
map_file	required	na	Density map of interest in CCP4 or MRC format.
model_file	required	na	Model corresponding to density map of interest.
threshold = <float>	required	na	Threshold value at which resolution appropriate features can be seen.
half1 = <map_file>	optional	na	Density half-map 1 in CCP4 or MRC format.
half2 = <map_file>	optional	na	Density half-map 2 in CCP4 or MRC format.
bandwidth_weight	optional	10	Bandwidth estimator for mean shift clustering. Values above 30 are required for higher resolution density maps.
model_dist	optional	5	Maximum distance (Å) from any atom in model; points beyond this are excluded.
half_thresh	optional	0.5	Threshold difference (in map sigma) at which voxels are filtered out.

**Table 3:** Modeling Results. A table summarizing the results for the three datasets from the 2021 CryoEM Ligand Challenge is shown. All statistics were taken directly from the model comparison pipeline (https://model-compare.emdataresource.org/2021/cgi-bin/index.cgi, accessed on 1 January 2022) or generated with Phenix. All scores are generated using the final, refined model with the exception of the TM-score, which was calculated from the probabilistic model. RMSDs are calculated using only C-alpha atoms. Modeling Results. A table summarizing the results for the three datasets from the 2021 CryoEM Ligand Challenge is shown. All statistics were taken directly from the model comparison pipeline (https://model-compare.emdataresource.org/2021/cgi-bin/index.cgi, accessed on 1 January 2022) or generated with Phenix. All scores are generated using the final, refined model with the exception of the TM-score, which was calculated from the probabilistic model. RMSDs are calculated using only C-alpha atoms.

Score	Beta-Galactosidase (Emd-7770)	RNA Polymerase (Emd-30210)	ORF3a Ion Channel (Emd-22898)
Map Resolution (Å)	1.9	2.5	2.1
Molprobity score	1.72	1.69	1.37
Clash score	5.78	2.97	5.75
HOH clash	2.49	1.88	0.0
Ramachandran Outliers	0.10	0.0	0.0
Rotamer outliers	1.35	3.14	1.18
FSC (0.5)	2.10	2.67	2.19
CC Mask	0.91	0.72	0.84
All atom inclusion	0.91	0.81	0.79
EMRinger score	6.43	3.39	4.47
Qscore			
Protein	0.81	0.72	0.79
Ligand	0.85	0.79	0.71
Water	0.85	0.84	0.86
LDDT	0.97	0.96	0.99
GDT_TS	99.90	99.61	100.00
TM-score	0.8898	0.9950	0.9980
C-alpha RMSD (Å), reference model PDB_ID	0.20, 6CVM	0.37, 7BV2	0.95, 7KJR

## Data Availability

Density maps and reference structures are available from the EMDataResource. Models for the 2021 CryoEM Ligand Challenge reported in this paper can be found at https://challenges.emdataresource.org/?q=2021-model-challenge, accessed on 1 January 2022. All software developed in this study is available free for academic use as part of the Phenix software suite. All other data supporting this publication are available from the corresponding author.

## Supplementary Materials

The following supporting information can be downloaded at https://www.mdpi.com/article/10.3390/biom12060773/s1, Figure S1: Comparison of pseudoatom generation methods; Figure S2: Comparison of TSP solvers. References [25,30,33,36,46,47,48] are cited in the supplementary materials.

## References

[1] Callaway E. (2020). Revolutionary Cryo-EM Is Taking over Structural Biology. Nature.

[2] Cressey D., Callaway E. (2017). Cryo-Electron Microscopy Wins Chemistry Nobel. Nature.

[3] Wigge C., Stefanovic A., Radjainia M. (2020). The Rapidly Evolving Role of Cryo-EM in Drug Design. Drug Discov. Today Technol..

[4] Egelman E.H. (2016). The Current Revolution in Cryo-EM. Biophys. J..

[5] Henderson R., Sali A., Baker M.L., Carragher B., Devkota B., Downing K.H., Egelman E.H., Feng Z., Frank J., Grigorieff N. (2012). Outcome of the First Electron Microscopy Validation Task Force Meeting. Structure.

[6] Lawson C.L., Kryshtafovych A., Adams P.D., Afonine P.V., Baker M.L., Barad B.A., Bond P., Burnley T., Cao R., Cheng J. (2021). Cryo-EM Model Validation Recommendations Based on Outcomes of the 2019 EMDataResource Challenge. Nat. Methods.

[7] Kišonaitė M., Wild K., Lapouge K., Ruppert T., Sinning I. (2022). High-Resolution Structures of a Thermophilic Eukaryotic 80S Ribosome Reveal Atomistic Details of Translocation. Nat. Commun..

[8] Liao M., Cao E., Julius D., Cheng Y. (2013). Structure of the TRPV1 Ion Channel Determined by Electron Cryo-Microscopy. Nature.

[9] Liang Y.-L., Khoshouei M., Radjainia M., Zhang Y., Glukhova A., Tarrasch J., Thal D.M., Furness S.G.B., Christopoulos G., Coudrat T. (2017). Phase-Plate Cryo-EM Structure of a Class B GPCR-G-Protein Complex. Nature.

[10] Lapinaite A., Knott G.J., Palumbo C.M., Lin-Shiao E., Richter M.F., Zhao K.T., Beal P.A., Liu D.R., Doudna J.A. (2020). DNA Capture by a CRISPR-Cas9–Guided Adenine Base Editor. Science.

[11] Yao H., Song Y., Chen Y., Wu N., Xu J., Sun C., Zhang J., Weng T., Zhang Z., Wu Z. (2020). Molecular Architecture of the SARS-CoV-2 Virus. Cell.

[12] Cheng Y. (2018). Single-Particle Cryo-EM-How Did It Get Here and Where Will It Go. Science.

[13] Nakane T., Kotecha A., Sente A., McMullan G., Masiulis S., Brown P.M.G.E., Grigoras I.T., Malinauskaite L., Malinauskas T., Miehling J. (2020). Single-Particle Cryo-EM at Atomic Resolution. Nature.

[14] Xie Q., Yoshioka C.K., Chapman M.S. (2020). Adeno-Associated Virus (AAV-DJ)-Cryo-EM Structure at 1.56 Å Resolution. Viruses.

[15] Yip K.M., Fischer N., Paknia E., Chari A., Stark H. (2020). Atomic-Resolution Protein Structure Determination by Cryo-EM. Nature.

[16] Zhang K., Pintilie G.D., Li S., Schmid M.F., Chiu W. (2020). Resolving Individual Atoms of Protein Complex by Cryo-Electron Microscopy. Cell Res..

[17] Saibil H.R. (2022). Cryo-EM in Molecular and Cellular Biology. Mol. Cell.

[18] Jiang W., Baker M.L., Ludtke S.J., Chiu W. (2001). Bridging the Information Gap: Computational Tools for Intermediate Resolution Structure Interpretation. J. Mol. Biol..

[19] Rossmann M.G. (2000). Fitting Atomic Models into Electron-Microscopy Maps. Acta Crystallogr. D Biol. Crystallogr..

[20] Tama F., Miyashita O., Brooks C.L. (2004). Flexible Multi-Scale Fitting of Atomic Structures into Low-Resolution Electron Density Maps with Elastic Network Normal Mode Analysis. J. Mol. Biol..

[21] Topf M., Baker M.L., John B., Chiu W., Sali A. (2005). Structural Characterization of Components of Protein Assemblies by Comparative Modeling and Electron Cryo-Microscopy. J. Struct. Biol..

[22] Baker M.L., Baker M.R., Hryc C.F., Dimaio F. (2010). Analyses of Subnanometer Resolution Cryo-EM Density Maps. Methods Enzymol..

[23] DiMaio F., Tyka M.D., Baker M.L., Chiu W., Baker D. (2009). Refinement of Protein Structures into Low-Resolution Density Maps Using Rosetta. J. Mol. Biol..

[24] Emsley P., Lohkamp B., Scott W.G., Cowtan K. (2010). Features and Development of Coot. Acta Crystallogr. D Biol. Crystallogr..

[25] Wang Z., Hryc C.F., Bammes B., Afonine P.V., Jakana J., Chen D.-H., Liu X., Baker M.L., Kao C., Ludtke S.J. (2014). An Atomic Model of Brome Mosaic Virus Using Direct Electron Detection and Real-Space Optimization. Nat. Commun..

[26] Hryc C.F., Baker M.L., Glaeser R.M., Nogales E., Chiu W. (2021). Model Building and Validation. Single-Particle Cryo-EM of Biological Macromolecules.

[27] Terwilliger T.C., Adams P.D., Afonine P.V., Sobolev O.V. (2018). A Fully Automatic Method Yielding Initial Models from High-Resolution Cryo-Electron Microscopy Maps. Nat. Methods.

[28] Terashi G., Kihara D. (2018). De Novo Main-Chain Modeling for EM Maps Using MAINMAST. Nat. Commun..

[29] Pfab J., Phan N.M., Si D. (2021). DeepTracer for Fast de Novo Cryo-EM Protein Structure Modeling and Special Studies on CoV-Related Complexes. Proc. Natl. Acad. Sci. USA.

[30] Baker M.R., Rees I., Ludtke S.J., Chiu W., Baker M.L. (2012). Constructing and Validating Initial Cα Models from Subnanometer Resolution Density Maps with Pathwalking. Structure.

[31] Chen M., Baker M.L. (2018). Automation and Assessment of de Novo Modeling with Pathwalking in near Atomic Resolution CryoEM Density Maps. J. Struct. Biol..

[32] Chen M., Baldwin P.R., Ludtke S.J., Baker M.L. (2016). De Novo Modeling in Cryo-EM Density Maps with Pathwalking. J. Struct. Biol..

[33] Helsgaun K. (2009). General K-Opt Submoves for the Lin–Kernighan TSP Heuristic. Math. Program. Comput..

[34] Barad B.A., Echols N., Wang R.Y.-R., Cheng Y., DiMaio F., Adams P.D., Fraser J.S. (2015). EMRinger: Side Chain-Directed Model and Map Validation for 3D Cryo-Electron Microscopy. Nat. Methods.

[35] Williams C.J., Headd J.J., Moriarty N.W., Prisant M.G., Videau L.L., Deis L.N., Verma V., Keedy D.A., Hintze B.J., Chen V.B. (2018). MolProbity: More and Better Reference Data for Improved All-Atom Structure Validation. Protein Sci. Publ. Protein Soc..

[36] Liebschner D., Afonine P.V., Baker M.L., Bunkóczi G., Chen V.B., Croll T.I., Hintze B., Hung L.W., Jain S., McCoy A.J. (2019). Macromolecular Structure Determination Using X-Rays, Neutrons and Electrons: Recent Developments in Phenix. Acta Crystallogr. Sect. Struct. Biol..

[37] Pettersen E.F., Goddard T.D., Huang C.C., Couch G.S., Greenblatt D.M., Meng E.C., Ferrin T.E. (2004). UCSF Chimera--a Visualization System for Exploratory Research and Analysis. J. Comput. Chem..

[38] Pettersen E.F., Goddard T.D., Huang C.C., Meng E.C., Couch G.S., Croll T.I., Morris J.H., Ferrin T.E. (2021). UCSF ChimeraX: Structure Visualization for Researchers, Educators, and Developers. Protein Sci. Publ. Protein Soc..

[39] Baker M.R., Fan G., Seryshev A.B., Agosto M.A., Baker M.L., Serysheva I.I. (2021). Cryo-EM Structure of Type 1 IP3R Channel in a Lipid Bilayer. Commun. Biol..

[40] Fan G., Baker M.R., Wang Z., Seryshev A.B., Ludtke S.J., Baker M.L., Serysheva I.I. (2018). Cryo-EM Reveals Ligand Induced Allostery Underlying InsP3R Channel Gating. Cell Res..

[41] Pintilie G., Zhang K., Su Z., Li S., Schmid M.F., Chiu W. (2020). Measurement of Atom Resolvability in CryoEM Maps with Q-Scores. Nat. Methods.

[42] Zhang Y., Skolnick J. (2004). Scoring Function for Automated Assessment of Protein Structure Template Quality. Proteins.

[43] Bartesaghi A., Aguerrebere C., Falconieri V., Banerjee S., Earl L.A., Zhu X., Grigorieff N., Milne J.L.S., Sapiro G., Wu X. (2018). Atomic Resolution Cryo-EM Structure of β-Galactosidase. Structure.

[44] Kern D.M., Sorum B., Mali S.S., Hoel C.M., Sridharan S., Remis J.P., Toso D.B., Kotecha A., Bautista D.M., Brohawn S.G. (2021). Cryo-EM Structure of the SARS-CoV-2 3a Ion Channel in Lipid Nanodiscs. bioRxiv.

[45] Yin W., Mao C., Luan X., Shen D.-D., Shen Q., Su H., Wang X., Zhou F., Zhao W., Gao M. (2020). Structural Basis for Inhibition of the RNA-Dependent RNA Polymerase from SARS-CoV-2 by Remdesivir. Science.

[46] Rotkiewicz P., Skolnick J. (2008). Fast Procedure for Reconstruction of Full-Atom Protein Models from Reduced Representations. J. Comput. Chem..

[47] Pedregosa F., Varoquaux G., Gramfort A., Michel V., Thirion B., Grisel O., Blondel M., Prettenhofer P., Weiss R., Dubourg V. (2011). Scikit-Learn: Machine Learning in Python. J. Mach. Learn. Res..

[48] Perron L., Furnon V. OR-Tools 7.2. https://developers.google.com/optimization.

