# Supplementary material for: Beyond the Backbone: The Next Generation of Pathwalking Utilities for Model Building in CryoEM Density Maps
Source: Biomolecules. 2022 Jun 2;12(6):773. doi: 10.3390/biom12060773 (PMC9220806; doi:10.3390/biom12060773)
Supplement: Supplementary file 1 [file biomolecules-12-00773-s001.zip › biomolecules-1722641-supplementary.pdf]

**Supplementary Methods**

In the current version of Pathwalking, we addressed all five major areas of Pathwalking, improving both usability and model accuracy (**Figure 1**). In integrating with the Phenix data structures, Pathwalking now has access to a range of map and model utilities that were not previously available. Many of these features have been directly incorporated into Pathwalking as options and are available through the command line or GUI (**Table 1**). Map filtration, the first step in the Pathwalking process, which helps to remove noise from the reconstruction and enhance map features, is now available as an option. Once a path is determined, Pathwalking can automatically thread the protein sequence onto the path, add all mainchain and sidechain atoms with Pulchra [46], and perform real-space refinement [25]. Threading of the sequence onto the Pathwalking model is performed in both directions, as the resulting trace is essentially directionless. From this threaded model, Pulchra, available through Phenix [36], allows for all-atom reconstruction and refinement from a reduced protein model, in this case, the C$\alpha$ backbone trace with sequence assignment from Pathwalking. Refinement of the model with respect to the density is also carried out through Phenix using the default options for real-space refinement. All of the aforementioned steps can be accomplished in the context of a single Pathwalking instance in Phenix, whereas in previous Pathwalking versions, these steps would have been accomplished with no less than four separate utilities.

Among the key algorithm developments in the latest version of Pathwalking is the inclusion of scikit-learn [47], a set of machine learning tools built with Python (https://scikit-learn.org/stable/). In the original version of Pathwalking [30], pseudoatom generation was accomplished using a modified k-Means clustering algorithm, whereby users supply a threshold and number of clusters (pseudoatoms) as inputs. With scikit-learn, we now support not only the k-Means clustering but have also included spectral clustering, Gaussian mixture models, agglomerative clustering, and mean-shift clustering (**Figure S1**). These new clustering algorithms provide alternate approaches to seeding the density map with pseudoatoms and can be useful in different resolution maps. The K- means method tends to give an even weight to density features, while spectral clustering appears to be very sensitive to noise and higher resolution features. Mean-shift clustering is particularly well-suited for low resolutions but tends to give very linear point distributions that distort secondary structure. Gaussian mixture models and agglomerative clustering perform very similarly to k- means clustering at near-atomic resolutions but also tend to favor sidechain density at higher resolutions. Regardless, each of these clustering algorithms provides very similar overall results in pseudoatom placement and requires comparable amounts of computing time.

Another significant algorithm improvement was the inclusion of Google's OR tools [48], which provides a Python-based TSP solver (https://developers.google.com/optimization). As a default, Pathwalking previously used the LKH TSP solver (http://webhotel4.ruc.dk/~keld/research/LKH/) [33], which is supported across various operating systems but must be downloaded and installed separately. Google's OR tools provide the same TSP functionality as LKH, with the addition of several path filtering options, and can be bundled with Phenix and Pathwalking, making it the ideal default TSP solver. The LKH and OR tools TSP solvers perform similarly (**Figure S2**), though the OR tools TSP solver compute time can be nearly 10 times that of the LKH solver (3sec vs 30sec). Despite the additional time, the more permissive licensing of OR tools and ability to bundle the software with Phenix has resulted in OR tools becoming the default TSP solver for Pathwalking.

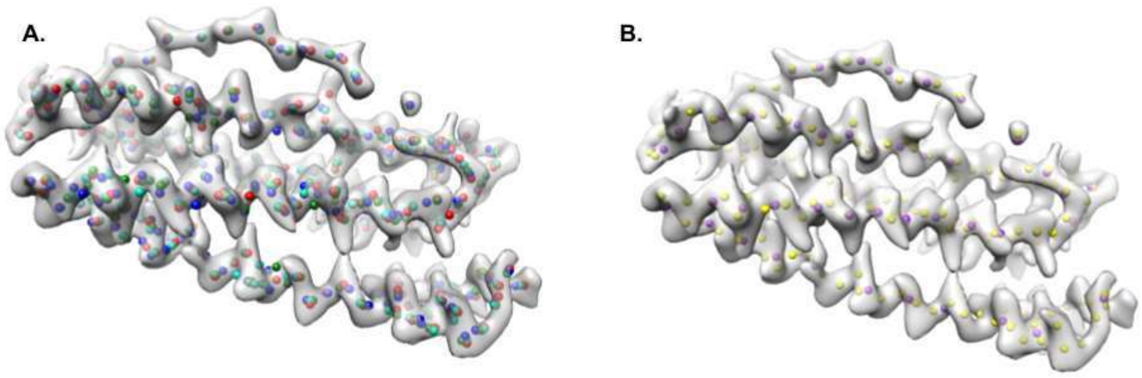


**Figure S1.** Comparison of pseudoatom generation methods. The currently supported methods of pseudoatom generation are shown on a segmented subunit of human apoferritin (EMDB 20028). In (**A**), pseudoatoms generated with k-means are shown in green, Gaussian mixture models in cyan, agglomerative clustering in navy, and spectral clustering in red. In (**B**), mean-shift clustering is shown in yellow and purple with a bandwidth of 2 and 3, respectively.

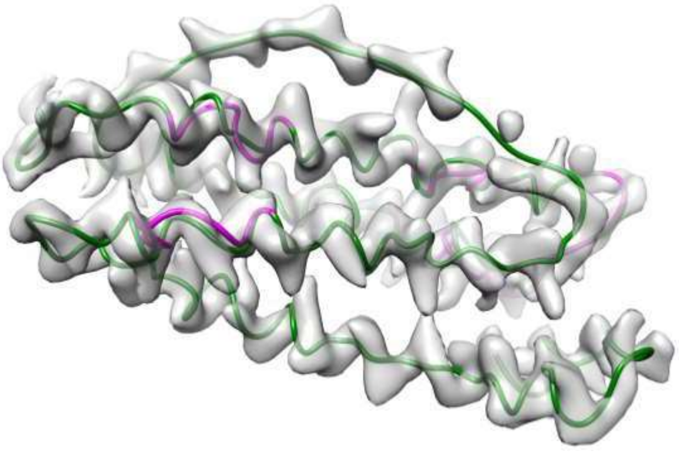

**Figure S2.** Comparison of TSP solvers. A full run of Pathwalking on a subunit of human apoferritin (EMDB 20028). The OR tools TSP solver results are shown in the green path, while the LKH solver path is shown in dark pink. The paths are nearly identical; the primary differences arise from a slight difference in the placement of pseudoatoms.
